# Successful treatment with BrECADD in a patient with Dubin-Johnson syndrome and classic Hodgkin lymphoma: a case report

**DOI:** 10.1007/s00280-026-04975-w

**Published:** 2026-10-23

**Authors:** Hishan Tharmaseelan, Christian Peter Jaworek, Peter Borchmann, Conrad-Amadeus Voltin, Martin Galler, Gareth J. Veal, Louisa Lepkes, Björn Reusch, Clara Velmans, Christian Netzer, Wenyu Yang, Uwe Fuhr, Shelby Barnett

**Affiliations:** 1 Faculty of Medicine and University Hospital of Cologne, Department I of Internal Medicine, and Center for Integrated Oncology Aachen Bonn Cologne Düsseldorf (CIO ABCD), German Hodgkin Study Group (GHSG), University of Cologne, Kerpener Str 62, 50937 Cologne, Germany; 2 Department of Nuclear Medicine, Faculty of Medicine and University Hospital Cologne, University of Cologne, Cologne, Germany; 3 Molecular Imaging and Therapy Service, Department of Radiology, Memorial Sloan Kettering Cancer Center, New York, NY USA; 4 Newcastle University Centre for Cancer, Newcastle University, Newcastle upon Tyne, UK; 5 Institute of Human Genetics, Faculty of Medicine and University Hospital Cologne, University of Cologne, Cologne, Germany; 6 Clinical Pharmacology, Department I of Pharmacology, Faculty of Medicine and University Hospital Cologne, University of Cologne, Cologne, Germany

**Keywords:** Dubin-Johnson syndrome, Classic Hodgkin lymphoma, ABCC2, BrECADD, Genetic polymorphisms, Therapeutic drug monitoring

## Abstract

Dubin-Johnson syndrome (DJS) results from ABCC2/MRP2 mutations causing impaired biliary transport. MRP2 also mediates hepatic elimination of chemotherapeutic agents, yet no data exist on anthracycline-based chemotherapy in adult DJS patients. We report a 26-year-old male with advanced-stage classic Hodgkin lymphoma and DJS harboring a novel homozygous ABCC2 variant (c.2291G>A, p.Gly764Asp). After a pembrolizumab prephase, full-dose BrECADD was administered, and samples were taken for therapeutic drug monitoring (TDM), which demonstrated normal etoposide and doxorubicin pharmacokinetics. Complete metabolic remission was achieved without significant toxicity. This case demonstrates that TDM-guided, full-dose anthracycline-based chemotherapy is feasible in DJS.

**Supplementary Information:**

The online version contains supplementary material available at https://doi.org/10.1007/s00280-026-04975-w.

Dubin-Johnson syndrome (DJS) is a rare (affects fewer than one per 100,000 individuals) autosomal recessive disorder caused by loss-of-function variants in the ATP Binding Cassette Subfamily C Member 2 (ABCC2) gene, encoding the canalicular transporter Multidrug Resistance–associated Protein 2 (MRP2) [1]. MRP2 mediates biliary excretion of organic anions, and its deficiency causes isolated conjugated hyperbilirubinemia without hepatocellular injury [2]. Importantly, MRP2 also participates in hepatic elimination of chemotherapeutic agents including platinum-based agents, vinca alkaloids, and anthracyclines [3]. Published clinical pharmacology data on chemotherapy in DJS are limited to a recent single pediatric case reporting threefold elevated vincristine exposure [4]. Thus, treatment in DJS patients must be closely monitored. No data exist on anthracycline-containing polychemotherapy in adult DJS patients. We report the first case of successful full-dose BrECADD (brentuximab vedotin, etoposide, cyclophosphamide, doxorubicin, dacarbazine, and dexamethasone) treatment with a corticosteroid and pembrolizumab prephase for advanced-stage classic Hodgkin lymphoma (cHL) in a patient with DJS and a novel homozygous *ABCC2* variant.

A 26-year-old male presented with B-symptoms, left axillary lymphadenopathy, and new-onset jaundice of approximately five weeks’ duration. His parents were first cousins, and his father had suspected DJS. Laboratory evaluation showed conjugated hyperbilirubinemia (total bilirubin 6 mg/dL; reference < 1.1) and elevated C-reactive protein (CRP) (156.2 mg/L). Liver transaminases were normal at presentation; alkaline phosphatase was minimally increased (158 U/L, reference range 40–129). An excisional biopsy confirmed cHL of the mixed cellularity type subtype with both CD30 expression and EBV (Epstein-Barr virus) positivity in immunohistochemistry. Baseline ^18^F-FDG-PET/CT demonstrated advanced-stage cHL with left axillary, mediastinal, splenic, and retroperitoneal involvement with a metabolic tumor volume (MTV) of 403 mL (detailed imaging is available in Supplementary Figure S2). In summary, the disease was in supra- and infradiaphragmatic, thus in advanced stage, with Ann Arbor stage IIIB, an increased Erythrocyte Sedimentation Rate (ESR) of 81 mm/h (ref < 11), and a Hasenclever International Prognostic Score of 2 (low albumine (34 g/L) and male sex).

The differential diagnosis of the new-onset jaundice included hepatic lymphoma infiltration, vanishing bile duct syndrome (VBDS; a paraneoplastic cholestatic condition associated with Hodgkin lymphoma [5]), hepatitis and DJS. Given the consanguinity, suspected paternal DJS, and conjugated pattern with normal liver enzymes, DJS was considered most likely, though VBDS could not be excluded at treatment initiation. Genetic analysis, performed in parallel but not yet available at the time of treatment, later showed a homozygous missense variant c.2291G > A;(p.Gly764Asp) in *ABCC2* exon 18 (rs2038470660) - a novel variant with pathogenic prediction (REVEL score 0.976). The variant is classified as likely pathogenic and therefore molecularly confirms DJS in the patient [ACMG criteria: PM2_supporting, PM3_supporting, PP3_strong] [6]. The segregation analysis of the parents by Sanger sequencing showed that the father is also homozygous for the variant and the mother is a heterozygous carrier (Supplementary Figure S2). This is in line with the clinical diagnosis of DJS in the father and provides further evidence for pathogenicity of this novel variant. This is to our knowledge the first described case with DJS and pseudodominant inheritance.

A standard treatment for advanced cHL is BrECADD [7]. However, impaired MRP2 function could alter the elimination of drugs including doxorubicin and etoposide [8], raising potential toxicity concerns with standard dose chemotherapy. While the pharmacokinetics of etoposide are unaltered by hyperbilirubinemia [9], a dose reduction for doxorubicin is recommended [10].

After a multidisciplinary discussion, we adopted a sequential strategy: an oral prephase for five days (prednisone 100 mg p. o.), followed by one cycle of intravenous pembrolizumab (200 mg) as an MRP2-independent cytoreduction to potentially rule out a paraneoplastic VBDS, and subsequent BrECADD with systematic therapeutic drug monitoring (TDM) of the plasma concentrations of potentially MRP2-mediated excreted drugs, i.e. etoposide (150 mg/m^2^ BSA; 307 mg absolute) and doxorubicin (40 mg/m^2^ BSA; 82 mg absolute).

Three weeks after a single cycle of pembrolizumab, ¹⁸F-FDG PET/CT staging was performed, which showed a MTV reduction (at SUV4.0 threshold) of 85% (from 403 to 58 mL), with simultaneously declining bilirubin (Fig. 1A and C). Consequently, polychemotherapy was initiated at standard doses and total plasma concentrations of etoposide and doxorubicin were quantified at the end of i.v. infusion (1 h for etoposide, 0.5 h for doxorubicin) and over a 72-hour period following drug infusion using a fully validated LC/MS/MS assay (Fig. 1B, Supplementary Figure S3). Pharmacokinetic results are presented in Table 1.

**Fig. 1:**
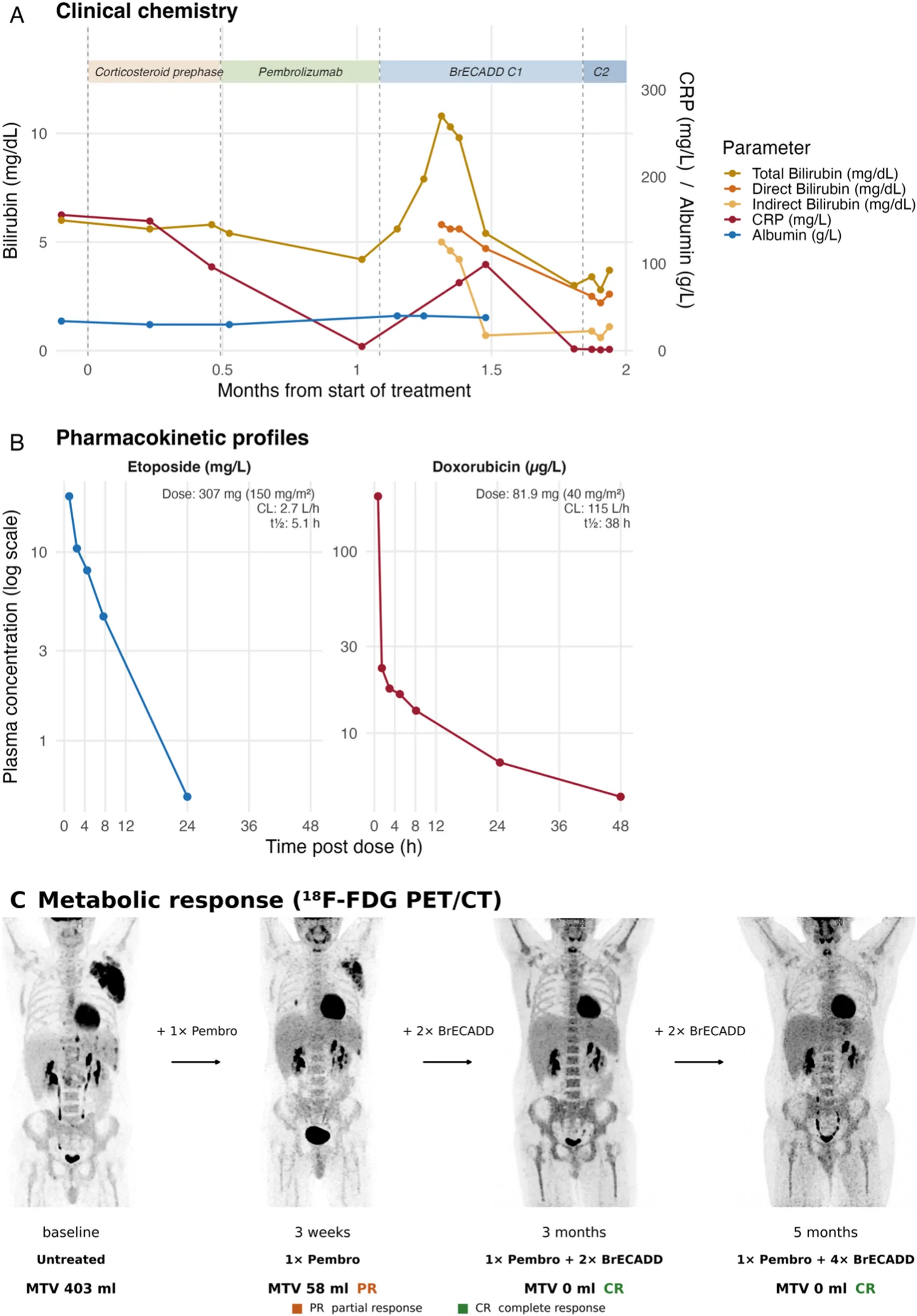
(**A**) Course of serum bilirubin (total, direct, indirect; left axis) with CRP and albumin (right axis, scaled) over the first months of treatment. Colored strip marks treatment phases. Filled circles: measured values; lines: linear interpolation. (**B**) Plasma concentration–time profiles (semi-log) of etoposide and doxorubicin after the first dose of BrECADD cycle 1. (**C**) Serial ^18^F-FDG-PET/CT MIP images demonstrating metabolic response to therapy: bulky baseline disease (MTV 403 mL) achieved partial metabolic remission and a massive reduction in tumor volume after a single cycle of pembrolizumab (MTV 58 mL, 85% reduction), and complete metabolic remission after the addition of two cycles of BrECADD (MTV 0 mL). CRP: C-reactive protein; C1/2: cycle 1/2

The extended TDM during the first BrECADD cycle demonstrated no impairment of elimination for either etoposide or doxorubicin [Table 1; Fig. 1B], permitting full-dose continuation. Following the first cycle of BrECADD febrile neutropenia occurred which was treated with empirical antibiotics without any pathogen detected. After one cycle of pembrolizumab and two cycles of BrECADD restaging ^18^F-FDG-PET/CT showed complete metabolic remission (MTV 0 mL) which was sustained after four standard dose cycles of BrECADD. The treatment was exceptionally well tolerated without additional clinically significant toxicity.

**Table 1:** Pharmacokinetic parameters of etoposide and doxorubicin

Pharmacokinetic parameter (1st dose)	Dose	AUC_0−∞_	Clearance	t_1/2_
Etoposide (this patient)	307 mg (150 mg/m^2^)	116 h* mg/L	2.7 L/h	5.1 h
Etoposide (examples for published data)	120 mg/m² ref. [12]	220–322 h * mg/L [12]	1.5–3.5 L/h [13]	4–11 h [13]
Doxorubicin (this patient)	82 mg (40 mg/m^2^)	711 h* µg/L	115 L/h	37.5 h
Doxorubicin (examples for published data)	60 mg/m^2^ ref. [14]	337–1673 h·* µg/L [14]	25–75 L/h [10, 15]	15–50 h [10, 15]

Areas under the concentration vs. time profiles for the first dose were calculated by the trapezoidal rule and extrapolated to infinity (AUC_0−∞_) by adding the last observed concentration divided by the terminal elimination constant which was obtained by log-linear regression of the final part of the profiles. Total clearance was calculated as dose divided by AUC_0−∞_. Terminal elimination half-life (t_1/2_) was calculated as ln(2) divided by the elimination constant

This is, to our knowledge, the first report of successful full-dose anthracycline-based polychemotherapy in an adult patient with DJS. Reassuringly, the systemic exposures measured for both doxorubicin and etoposide did not exceed the ranges reported for adult cancer patients receiving these agents at conventional, body surface area-based doses (Table 1), despite pharmacokinetic parameters of these drugs being characterized by pronounced interindividual variability. It was also reassuring that the calculated drug elimination half-lives in our patient did not indicate prolonged elimination of the drugs which may be related to accumulation and/or toxicity.

The pharmacokinetic profiles and drug exposures observed following TDM suggest that the missense variant c.2291G > A in *ABCC2* does not critically impair elimination of etoposide or doxorubicin at standard doses, contrasting with prior reports of markedly elevated vincristine levels in a pediatric DJS patient [4]. This may be explained by the drug-specific extent of MRP2 contribution to overall elimination, which is limited for etoposide and doxorubicin, along with variant-specific differences in residual MRP2 function. Whether this reflects variant-specific differences in residual MRP2 function, drug-specific transport activity, or age-related pharmacokinetic factors remains to be elucidated. Of interest, and supporting the findings of the current report, a normal pharmacokinetic profile and drug exposure were also observed following a standard dose of doxorubicin in the previously reported DJS patient experiencing excessive vincristine exposure [4].

Our approach illustrates a pragmatic strategy for delivering chemotherapy to patients with unknown pharmacological susceptibility and unclear hyperbilirubinemia: (1) a non-hepatically cleared agent (pembrolizumab) as a cytoreductive bridge, (2) systematic TDM to detect altered drug exposure, and (3) readiness for dose modification. The prephase with corticosteroids and checkpoint inhibitors thus served a dual purpose: tumor cytoreduction and *ex juvantibus* exclusion of VBDS or liver infiltration, which would respond to anti–PD-1 therapy only if driven by cHL.

The novel homozygous variant c.2291G > A;(p.Gly764Asp) in exon 18 adds to the known mutational spectrum of *ABCC2-*related DJS [11]. Its high REVEL score supports impaired function, though functional characterization of its impact on MRP2 transporter activity is pending. The genetic heterogeneity of DJS underscores that our findings may not generalize to all (likely) pathogenic *ABCC2* variants, and TDM along with clinical monitoring remains advisable when administering MRP2-substrate chemotherapy to DJS patients regardless of genotype.

Limitations include the single-case design and absence of functional transporter assays. Strengths include prospective TDM integration, complete documentation of the treatment course from diagnosis through confirmed remission, and characterization of a novel variant.

In conclusion, full-dose BrECADD chemotherapy is feasible in advanced cHL with concurrent DJS. However, given the heterogeneous genetic background of DJS, we recommend TDM for a rational and individualized framework and safe administration of MRP2-substrate chemotherapy in this rare clinical setting.

## Supplementary Information

Below is the link to the electronic supplementary material.

Supplementary Material 1

## Data Availability

No datasets were generated or analysed during the current study.
