# Supplementary material for: Successful treatment with BrECADD in a patient with Dubin-Johnson syndrome and classic Hodgkin lymphoma: a case report
Source: Cancer Chemother Pharmacol. 2026 Oct 23;96(1):123. doi: 10.1007/s00280-026-04975-w (PMC13694284; doi:10.1007/s00280-026-04975-w)
Supplement: Supplementary file 1 [file 280_2026_4975_MOESM1_ESM.docx]

**Supplementary Material S1:**

**Supplementary Figure 1:** (A) Pedigree showing pseudodominant segregation of the *ABCC2* variant NM_000392.5:c.2291G>A, p.(Gly764Asp). The index patient (arrow) and the father are homozygous for the variant, whereas the mother is a heterozygous carrier; two clinically unaffected siblings were not genetically tested. Consanguinity is indicated by a double line. Mut, variant allele; Wt, reference allele. (B) Sanger sequencing electropherograms confirming the homozygous variant in the index patient and father and the heterozygous state in the mother. Coding DNA coordinates are shown below the traces, and the position of c.2291 is highlighted.

**Supplementary Figure S2:** Cross-sectional imaging highlighting malignant lesions (fused PET-CT imaging)

**Supplementary Figure S3: Assay reference information:**

- Etoposide – LLOQ 0.1µg/mL, linear range of assay: 0.1-10µg/mL.
- Doxorubicin – LLOQ 5.0ng/mL, linear range of assay: 5-500ng/mL.

Both assays are fully validated according to EMA and FDA guidelines, with inter- and intra-assay accuracy and precision within 15% across the calibration range for both assays.

References:

Veal GJ, Cole M, Errington J, Pearson AD, Gerrard M, Whyman G, Ellershaw C, Boddy AV. Pharmacokinetics of carboplatin and etoposide in infant neuroblastoma patients. Cancer Chemother Pharmacol. 2010 May;65(6):1057-66. doi: 10.1007/s00280-009-1111-9. Epub 2009 Aug 23. PMID: 19701749.

Mohmaed Ali MI, Nijstad AL, Boosman RJ, Crombag MBS, Barnett S, Veal GJ, Lalmohamed A, van Erp NP, Steeghs N, Zwaan CM, Beijnen JH, Siebinga H, Huitema ADR. A Population Pharmacokinetic Study to Evaluate Doxorubicin Exposure Across All Age Groups. Clin Pharmacokinet. 2024 Dec;63(12):1711-1722. doi: 10.1007/s40262-024-01445-5. Epub 2024 Nov 16. PMID: 39549227.
